# The London and Provincial Medical Directory, 1847

**Published:** 1847-04-01

**Authors:** 


The London and Provincial Medical Directory, 1847. pp. 600. Churchill.

This, now conjoined work, promises to be of great utility to the profession. The proprietors, we believe, spared neither pains or expense to acquire accurate information, and, as far as the metropolis is concerned, the Directory may be regarded as nearly perfect. If it is, however, to be made an annual paying publication the price must be reduced, which may be easily accomplished by leaving out the immense proportion of matter utterly irrelevant to the true purposes of a directory, and by employing abbreviations, which now seem studiously avoided. In this way a half-crown annual might be easily constructed, which would be purchased, not by a few hundreds, but by many thousands. But for this, the platitudes of the anonymous *arbiter elegantium* concerning medical etiquette, the ridiculous self-chronicling of works, papers, or other forgotten literary claims to notice, and in fact all extraneous matter whatever, save that which as advertisements pays for its place, must be swept away; and a simple announcement of name, address, and qualification alone retained.

At the end of the work an analysis of its contents, giving something like a synoptical view of the existing classification of the profession might be given. Upon a small scale we have attempted this as regards the metropolis portion of the Directory; and find it furnishes the following numbers: *Consulting Physicians* 301, of whom 30 practise as *Physician-Accoucheurs: Physicians* holding legitimate degrees, but yet engaging in *general practice* also, 133: *Consulting Surgeons,* including the leading dentists, 176: Practitioners only holding the *College of Surgeons' Diploma,* 468: Practitioners only holding the *Apothecaries' License,* 275: Practitioners holding both *license* and *diploma,* 990. Besides these, 70 practitioners have returned their names as practising before 1815. We have not counted the Erlangen, Giessen, &c. degrees, but referred their holders to their English qualifications. These numbers give us a total of 2413 authorized practitioners, besides which there is a list of more than 360 persons (!) who have returned no qualification, and who, it is but fair to suppose, possess none. We have not analyzed the country list, believing it incomplete; but we make the sum total of its names amount to 8,286, which added to those of London, gives 10,699 for England and Wales.

